# Metastatic melanoma: An unusual cause of gastrointestinal bleeding and intussusception—A case report

**DOI:** 10.1016/j.ijscr.2018.10.057

**Published:** 2018-10-29

**Authors:** Sílvia Silva, Nádia Tenreiro, Ana Melo, José Lage, Herculano Moreira, Fernando Próspero, Paulo Avelar

**Affiliations:** Department of General Surgery, Centro Hospitalar Trás-os-Montes e Alto Douro, Avenida da Noruega, Lordelo, 5000-508, Vila Real, Portugal

**Keywords:** Malignant melanoma, Melanoma metastasis, Intussusception, Small bowel bleeding, Small bowel obstruction, case report

## Abstract

**Introduction:**

Malignant melanoma is responsible for 1–3% of all malignancies being the gastrointestinal tract one of the most common metastatic locations.

**Presentation of case:**

We present the case of a 71-year old male with previous history of cutaneous melanoma. Seven years later, while being studied due to suspected small bowel bleeding, he developed small bowel obstruction after being submitted to double balloon enteroscopy. Further investigation revealed small bowel intussusception. He was taken up for emergency laparotomy that confirmed ileal intussusception secondary to an intraluminal mass. We performed segmental enterectomy with primary anastomosis. Histology confirmed intestinal melanoma metastasis.

**Discussion:**

Malignant melanoma frequently spreads to the gastrointestinal tract. The presentation is mainly asymptomatic and the diagnosis is often made only after complications develop. Small bowel intussusception and gastrointestinal bleeding are unusual presentations of metastatic melanoma, with few cases reported. Surgical resection not only provides symptom control but also positively affects prognosis.

**Conclusion:**

Although usually asymptomatic, melanoma metastasis should always be considered in a patient with intestinal symptoms or chronic anemia and personal history of melanoma. Surgical resection is the treatment of choice, leading to an increase in overall survival.

## Highlights

• Malignant melanoma frequently spreads to the gastrointestinal tract (60%).
• Only 1–4% of melanoma metastases to the gastrointestinal tract are detected before death because most patients are asymptomatic.
• Gastrointestinal spread of previously treated malignant melanoma should always be considered in patients with digestive symptoms or ferropenic anemia.
• Small bowel intussusception and gastrointestinal bleeding are unusual presentations of intestinal melanoma metastases.
• Surgical resection remains the mainstay of treatment, not only providing symptomatic control but also leading to improved survival.

## 1 Introduction

Malignant melanoma is responsible for 1–3% of malignant disease [1]. It frequently spreads to the gastrointestinal tract, with 60% of patients with advanced metastatic disease showing digestive involvement [[2], [3], [4], [5]]. Small bowel is the most common location of melanoma metastases in the gastrointestinal tract (GI) [1,2,6].

Symptoms of small intestinal involvement are frequently unspecific (abdominal pain, nausea, vomiting, weight loss and weakness) which leads to a late diagnosis often made only after complications [[7], [8], [9]]. The most common complications are intestinal obstruction, massive gastrointestinal bleeding and perforation [9].

We present the case of a patient with a unique array of symptoms secondary to metastatic malignant melanoma, with a combination of two possible complications of GI spread.

This case report was written according to SCARE guidelines [10].

## 2 Presentation of case

We present the case of a 71-year-old Caucasian male with previous medical history of type 2 diabetes and arterial hypertension. He also had personal history of superficial spreading melanoma of the lower limb treated with surgical excision in another institution 7 years before. It was performed surgical excision with margins of 1 cm. It was a stage IA tumor according to 2002 American Joint Committee on Cancer (AJCC) stage groupings for cutaneous melanoma [11]. Sentinel lymph node biopsy was not performed and no adjuvant treatment was made.

During routine blood analysis it was revealed ferropenic anemia that led to upper and lower endoscopy, with no evidence of bleeding, and capsule endoscopy that showed an ulcerated distal ileal lesion. He was admitted for double balloon enteroscopy with biopsy. After the exam he complained of abdominal pain and distension pain, and admitted inability to pass flatus or stool in the previous 4 days.

On physical exam he was pale, apyretic and hemodynamically stable. Abdomen was distended with hyperactive bowel sounds. Palpation was painful but without rebound tenderness. A 5 cm painless mass was palpable on left groin.

**Fig. 1:**
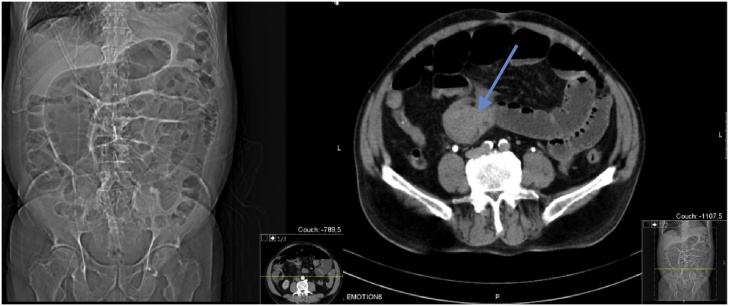
Preoperative computed tomography scan showing small bowel mass (arrow) causing intestinal dilatation and obstruction.

**Fig. 2:**
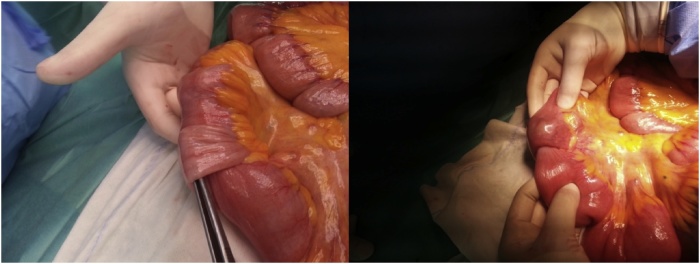
Intraoperative findings demonstrating ileal intussusception.

**Fig. 3:**
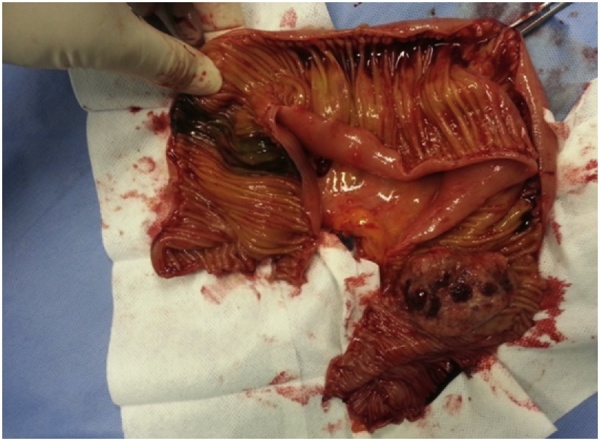
Intraoperative finding of an exophytic pigmented tumor.

Standard abdominal computed tomography (CT) scan showed dilatation of the small bowel and an area of bowel-within-bowel configuration in the distal ileum suggesting intussusception (Fig. 1). We performed an exploratory laparotomy, confirming the presence of small bowel intussusception (Fig. 2) with the lead point being an intraluminal intestinal mass (Fig. 3). The aforementioned inguinal mass appeared to be a lymph node conglomerate. We performed a segmental enterectomy with primary anastomosis and inguinal lymph node excisional biopsy. Histopathologic examination of small bowel and left inguinal lymph node mass was compatible with metastatic melanoma.

Post-operative period was uneventful and the patient was discharged on the 6th postoperative day.

He was referred to a melanoma oncological center where he underwent adjuvant chemotherapy with dacarbazine, bilateral extended inguinal lymphadenectomy and inguinal radiotherapy (60 Gy/30 F).

Sixteen months after, follow-up abdominal CT scan identified progression of disease in inguinal and retroperitoneal lymph nodes. He was restarted on chemotherapy with dacarbazine and six months later initiated immunotherapy with anti PD-1 agent Nivolumab, due to absence of response. Unfortunately, he maintained disease progression, with inguinal, retroperitoneal and supraclavicular nodal disease. At this stage the patient was referred to a Palliative Care consultation for support treatment.

He died 6 months later, 31 months after intestinal metastasis resection surgery and 7 years and 7 months after the first surgery.

## 3 Discussion

Malignant melanoma frequently spreads to the gastrointestinal tract and, among affected patients, the proportion with involvement of the small bowel ranges from 35% to 70% [1]. Superficial spreading melanoma is the most common histologic subtype and the most likely to metastasize to the small bowel [9].

Most patients with metastatic intestinal melanoma are asymptomatic and only 1–4% of metastases to the gastrointestinal tract are detected before death [[2], [3], [4], [5],^12^]. In these cases, diagnosis is often made after a complication develops [1].

The time frame period between diagnosis of primary malignant melanoma and the identification of metastases at a gastrointestinal site varies between 2 and 180 months [1,[13], [14], [15]]. In our case the time between surgical excision of primary tumor and small bowel metastasis identification was 84 months, which is in agreement.

Although malignant melanoma can metastasize to any digestive segment, the most common sites are the small bowel (51–71%), stomach (27%) and colon (22%) [6].

Gastrointestinal spread of malignant melanoma should be considered in patients with digestive symptoms or ferropenic anemia, requiring a directed endoscopic and radiological research [3].

The literature has described the usefulness of abdominal CT scan in the diagnosis of melanoma metastases to the small bowel, with an estimated sensitivity of about 66% [16]. This is confirmed by the fact that the diagnosis of small bowel melanoma metastases is mostly made post mortem [[2], [3], [4], [5],^12^].

Small bowel intussusception is a rare cause of intestinal obstruction in adult population, and is caused by neoplasia in 65% of cases [12]. However, intestinal melanoma metastasis as leading point to the intussusception is rarely reported in the literature [12]. Gastrointestinal bleeding as melanoma metastasis presentation is also an unusual condition [8].

The authors thus present a rare case of ileal intussusception and digestive bleeding secondary to ileal melanoma metastasis in a patient with primary cutaneous lesion excised seven years before.

Complete surgical resection of metastatic disease can provide important survival benefit. Gutman et al reported that the indications for surgery both elective and emergency had no impact on post-operative survival [17]. Ollila et al reported that median survival period after complete surgical resection of gastrointestinal metastases was 48.9 months while only 5.4 months after incomplete resection, and the 5-year survival rate was 41% after complete resection [18]. Branum et al also reported significantly longer survival after complete resection of gastrointestinal metastases than after incomplete resection, the mean survival period being 31.6 months versus 9.6 months [19]. In our case the resection was complete and the survival period was 31 months, which is in agreement with the literature.

Even when curative surgery is impossible because of the extent of the disease, gastrointestinal metastatic tumor resection is recommended to relieve symptoms or avoid future complications [13].

Standardized systemic therapy is lacking. Treatment of metastatic disease include chemotherapy and immunotherapy [15]. They can also be useful as a palliative treatment in metastatic intestinal melanoma but at their role is still unclear [20].

## 4 Conclusion

Diagnosis of gastrointestinal metastases of malignant melanoma is often late and in patients who undergo emergency surgery. Because of the high incidence of gastrointestinal metastases in patients with previous history of cutaneous melanoma and abdominal pain and/or anemia, modern imaging techniques are recommended in order to obtain an early diagnosis.

Surgical resection remains the mainstay of treatment in patients with resectable metastatic intestinal melanoma, not only providing symptomatic control but also leading to improved survival.

In the future, the evolving role of immunotherapy and genetically targeted treatment of metastatic malignant melanoma may further extend survival after surgical treatment.

## Conflicts of interest

Nothing to state.

## Funding

This research did not receive any specific grant from funding agencies in the public, commercial, or not-for-profit sectors.

## Ethical approval

Not submitted to ethical approval – Case report.

## Consent

Written informed consent was obtained from the patient daughter for publication of this case report and accompanying images.

## Author contribution

Sílvia Silva: study design, data collection, interpretation and writing.

Herculano Moreira: study concept, design and review of manuscript.

Nádia Tenreiro, Ana Melo: data collection and interpretation.

José Lage, Fernando Próspero, Paulo Avelar: review of manuscript.

## Registration of research studies

Not applicable.

## Guarantor

Herculano Moreira.

## Provenance and peer review

Not commissioned, externally peer reviewed.
